# Scalable Processing of Cyclic Olefin Copolymer (COC) Microfluidic Biochips †

**DOI:** 10.3390/mi14101837

**Published:** 2023-09-27

**Authors:** Rodolfo G. Rodrigues, Pedro G. M. Condelipes, Rafaela R. Rosa, Virginia Chu, João Pedro Conde

**Affiliations:** 1 Instituto de Engenharia de Sistemas e Computadores—Microsistemas e Nanotecnologias (INESC MN), Rua Alves Redol 9, 1000-029 Lisbon, Portugal; rodolfo.rodrigues@inesc-mn.pt (R.G.R.); pmonteiro@inesc-mn.pt (P.G.M.C.); rafaela.rosa@inesc-mn.pt (R.R.R.); vchu@inesc-mn.pt (V.C.); 2 Department of Bioengineering, Instituto Superior Técnico, Universidade de Lisboa, Av. Rovisco Pais 1, 1049-001 Lisbon, Portugal

**Keywords:** microfluidics, cyclic olefin copolymer (COC), polydimethylsiloxane (PDMS), hot embossing, thermal bonding, contact angles, molecular diffusion

## Abstract

Microfluidics evolved with the appearance of polydimethylsiloxane (PDMS), an elastomer with a short processing time and the possibility for replication on a micrometric scale. Despite the many advantages of PDMS, there are well-known drawbacks, such as the hydrophobic surface, the absorption of small molecules, the low stiffness, relatively high cost, and the difficulty of scaling up the fabrication process for industrial production, creating a need for alternative materials. One option is the use of stiffer thermoplastics, such as the cyclic olefin copolymer (COC), which can be mass produced, have lower cost and possess excellent properties. In this work, a method to fabricate COC microfluidic structures was developed. The work was divided into process optimization and evaluation of material properties for application in microfluidics. In the processing step, moulding, sealing, and liquid handling aspects were developed and optimized. The resulting COC devices were evaluated from the point of view of molecular diffusion, burst pressure, temperature resistance, and susceptibility to surface treatments and these results were compared to PDMS devices. Lastly, a target DNA hybridization assay was performed showing the potential of the COC-based microfluidic device to be used in biosensing and Lab-on-a-Chip applications.

## 1. Introduction

Microfluidic systems consist of microfabricated structures with feature sizes between the millimetre and nanometre scale, handling fluid volumes in the micro to femtolitre scales [1,2,3]. The first microfluidic devices were mainly used for analytical chemistry but currently they are used in many other areas, such as chemical synthesis and biomedical research in organ-on-a-chip systems [4]. The use of microfluidics has led to the creation of advanced platforms with multiple components used for different types of analysis [4,5]. The advantages of microfluidic systems for biological and analytical applications are low reagent consumption, fluid control, high surface-to-volume ratios, high throughput, rapid prototyping, and portability [5,6].

To develop a microfluidic device, it is necessary to use microfabrication methods that reduce the complexity of the process and its cost [7,8]. Additionally, for commercialization, microfabrication must be scalable to large numbers of devices. The first microfluidic devices were made of silicon or glass using techniques employed in the fabrication of microsystems and semiconductors [9,10]. In microfluidics, these materials were often used for devices for capillary electrophoresis, droplet formation, and chemical synthesis [11]. Although silicon and glass presented advantages for microfluidics, namely highly controllable surfaces, non-permeability to gases, and high stiffness, their fabrication costs were too high [12], which led to the search for alternative materials. Polymers are promising alternatives because they are low-cost, easy to process, and have a variety of available properties that can satisfy diverse system operating conditions [13].

The most common polymer used in microfluidics is polydimethylsiloxane (PDMS), an elastomer that has become the standard in lab-on-a-chip biodevices because it overcomes many limitations imposed by standard microfabrication techniques, namely complex channel formation and high-temperature sealing [14]. The fabrication process is known as soft lithography and was developed by the Whitesides group [15]. The soft lithography process consists of casting the PDMS on a master mould, sealing the cast, which is a negative of the mould, against a PDMS membrane or a glass substrate, and performing the connections by simply punching the PDMS with a needle.

Despite the widespread use of PDMS in microfluidics for biological applications, this technology has some limitations, such as low stiffness, high gas permeability, and its hydrophobic surface, which is a limitation in the case of capillary applications and may result in unwanted molecular adsorption [14,16]. Another limitation of PDMS soft lithography is the difficulty of large-scale production, which is time-consuming and user-dependent [17]. These limitations led to the study of other materials, such as thermoplastics. Thermoplastics are known for their ability to reshape after heating [3,11] and are already used in microfluidics for several applications [18]. The thermoplastic cyclic olefin copolymer (COC) is a viable alternative to PDMS, with properties such as low autofluorescence, wide transmittance range, thermal resistance, and biocompatibility [19,20]. Currently, its fabrication differs from that of PDMS, requiring high temperatures for hot embossing and injection moulding [11,21] and to the need to use thermal and solvent bonding methods to seal the device [11,22]. Several microfluidic applications have already been studied in COC, such as DNA amplification [22], cell culture chips [17], protein crystallization, and droplet formation [23].

In this work, we propose a scalable process to fabricate microfluidic systems in COC that does not require a heating ramp before the embossing of COC, as compared to other methods described in the literature [23,24,25]. A comparison between the hot embossing method described in this paper and the ones found in the literature is shown in Table 1. This device is sealed against a substrate of the same type of COC by thermal diffusion, without the need to use solvents or other functionalization methods. The type of COC used also has a glass transition temperature of 142 °C which can be used for high-temperature applications. Commercial polyether ether ketone (PEEK) connectors are used for fluid connection to the external appliances. Using this process, it is possible to replicate microstructures with minimum dimensions ranging from 20 to 150 microns in 10 min at process temperatures of 160 °C and process pressures of 2.3 MPa. Using this method, it was possible to fabricate up to six devices at the same time. Besides the development of a process for rapid prototyping of COC devices for microfluidic applications, relevant properties of the COC for microfluidic applications were also tested and compared to those of PDMS microfluidic devices. These tests included diffusion tests to observe if small molecules diffuse into the material, burst pressure tests, temperature tests to access the devices for applications that require different temperatures, the effect of surface treatments, and a target DNA hybridization assay.

## 2. Materials and Methods

### 2.1. Microfluidic Structure Fabrication

#### 2.1.1. Hard Mask Fabrication

The hard mask fabrication initiates with the design of the desired structure using AutoCAD 2023.1.2 software (Autodesk Inc., Mill Valley, CA, USA). Two hard masks with different patterns were fabricated, one for 100 µm and another for 20 µm height features.

Before the process starts, glass substrates (Corning Inc., Corning, NY, USA) were cleaned with isopropanol (IPA) (IPA 99.9%, LabChem Inc., Zelienople, PA, USA) and deionized water (DI water), followed by immersion in an Alconox solution (Alconox Inc., White Plains, NY, USA) for 30 min at 65 °C. The substrate was washed again with DI water and dried with compressed air. After the wash, a thin aluminium film of 200 nm thickness was deposited on a glass substrate by magnetron sputtering (Nordiko 7000, Nordiko Technical Services Ltd., Havant, Hampshire, UK). A Silicon Valley Group resist-coater track (Silicon Valley Group Inc., San Jose, CA, USA) was used to spin-coat 1.5 µm of a positive photoresist (PFR 7790G, JSR Micro, Inc., Sunnyvale, CA, USA) on top of the previously deposited aluminium layer and the photoresist was baked at 85 °C for 1 min. The pattern was transferred to the photoresist using a Heidelberg direct write laser (405 nm) lithography equipment (Heidelberg Instruments, Heidelberg, Germany). The photoresist was then baked at 110 °C for 1 min, cooled for 30 s, and developed for 1 min in TMA 238 WA (JSR Micro, Inc., Sunnyvale, CA, USA). The developer solution removed the photoresist from the areas previously exposed. The exposed aluminium was etched with an aluminium etchant (TechniEtch A180, Microchemicals, Ulm, Germany) until the exposed regions were completely removed. The remaining photoresist was removed with acetone (Acetone 99.6%, LabChem Inc., Zelienople, PA, USA), and IPA, completing the hard mask fabrication.

#### 2.1.2. SU-8 Mould Fabrication

The SU-8 mould was fabricated using the two aluminium hard masks described in Section 2.1.1. Silicon substrates (150 mm diameter, single side polished) were washed with acetone, IPA, and DI water, followed by immersion in an Alconox solution for 30 min at 65 °C. The substrate was washed again with DI water, dried with compressed air, and placed in an ultraviolet/ozone (UVO) cleaner (UVO-Cleaner^®^ 144X-220, Jelight Company Inc., Irvine, CA, USA) for 15 min. The fabrication process of the first mould layer, with 20 µm height, started with the spin-coating (WS-650MZ-23NPP/LITE spin coater, Laurell Technologies Corp., North Wales, PA, USA) of a negative photoresist (SU-8 2015, Microchem Corp., Newton, MA, USA). Spin-coating is a two-step process, beginning with 10 s at 500 rpm and 100 rpm/s followed by a second step of 34 s at 1700 rpm and 300 rpm/s. After the SU-8 coating, the photoresist was baked on a hot plate (Stuart, Staffordshire, UK) at 95 °C for 4 min and cooled down for 1 min. The first hard mask was placed over the SU-8 photoresist with the aluminium side towards the photoresist and exposed to ultraviolet light (UV light (254 nm, 400 W), UV Light Technology Limited, Birmingham, UK) for 30 s. The substrate was baked at 95 °C for 5 min and cooled down for 2 min. In the last step, the exposed SU-8 was developed in a propylene glycol monomethyl ether acetate (PGMEA) solution (PGMEA 99.5%, Sigma-Aldrich, St. Louis, MO, USA), rinsed with IPA, and dried with compressed air. This completed the fabrication of the first features in the SU-8 mould.

The process for the 100 µm thick layer was performed on top of the patterned photoresist from the previous step. It started with the spin coating of a negative photoresist (SU-8 50, Microchem Corp., Newton, MA, USA) for 10 s at 500 rpm and 100 rpm/s followed by 34 s at 1000 rpm and 300 rpm/s. After the coating, the sample was heated at 65 °C for 10 min, then the temperature was increased to 95 °C and, when this temperature was reached, the substrate stayed at this temperature for 30 min before being cooled for 1 min. The second hard mask was aligned with the photoresist, exposed to UV light for 70 s and baked at 65 °C for 1 min. The substrate with the new layer of photoresist was heated until 95 °C and left at this temperature for 10 min. The substrate was then cooled down for 2 min, developed in PGMEA solution for 10 min with manual agitation, washed with IPA and dried with compressed air. Lastly, the substrate went through a hard bake process at 150 °C for 15 min, finalizing the two-level SU-8 mould fabrication.

#### 2.1.3. PDMS Structure Fabrication

The process started with the mixture of curing agent and PDMS base (SYLGARD™ 184 Silicone Elastomer Kit, Dow Corning, Midland, MI, USA) in a 1:10 (*w*/*w*) ratio and the deaeration of this mixture in a desiccator (F42025-0000, Bel-Art Products, Inc., Warminster, PA, USA) for 45 min.

The PDMS mixture was poured into a poly(methyl methacrylate) (PMMA) (Perspex^®^ Cast Acrylic Sheet Clear, 3A Composites GmbH, Sins, Switzerland) frame containing the SU-8 mould described in 2.1.2. fixed at the bottom. Afterwards, the frame is closed and placed in the oven (Memmert oven 100–800, Memmert, Schwabach, Germany) to cure at 70 °C for 90 min. The cured PDMS is removed from the frame and peeled from the SU-8 mould, obtaining a positive PDMS structure. The positive PDMS structure is used for the fabrication of the epoxy mould and for the experimental tests.

For the experimental tests, the PDMS structure was punched in the inlet and outlet holes using a rounded 20 gauge needle (LS20, Instech Laboratories, Inc., Plymouth Meeting, PA, USA) to allow the connection between the structures and any external device. A 500 µm thick PDMS slab was fabricated using a spin-coating process for 25 s at 250 rpm and cured in the oven at 70 °C for 90 min. After that, the slab and the PDMS structure were placed in an oxygen plasma cleaner (Expanded Plasma Cleaner PDC-002, Harrick Plasma, Ithaca, NY, USA) for 1 min at 30 W and were sealed against each other.

#### 2.1.4. Epoxy Mould Fabrication

The liquid epoxy (Permabond^®^ ES562, Permabond Engineering Adhesives Ltd., Hampshire, UK) was poured on top of a positive PDMS structure that was previously fixed in a PMMA frame. The ensemble was placed at 50 °C for 60 min under vacuum. After the deaeration, the epoxy was cured at 120 °C for 30 min, obtaining the negative mould used for embossing.

#### 2.1.5. Embossing and De-Embossing of COC

**Figure 1:**
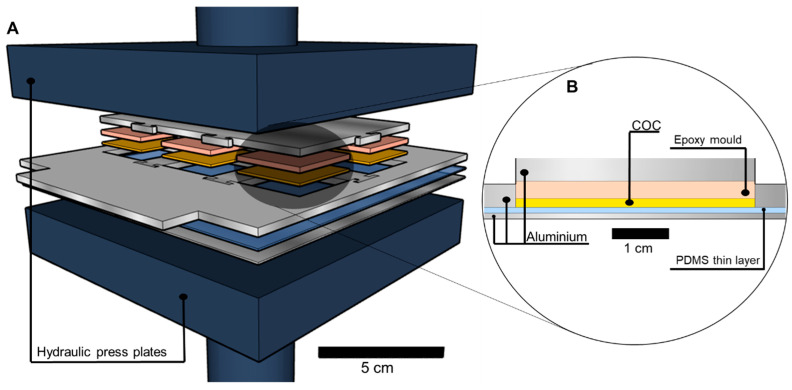
Schematic representation of COC processing for microfluidics: (**A**) hydraulic press representation and (**B**) stack used for the hot embossing.

The COC substrates (mcs-COC-13, microfluidic ChipShop GmbH, Jena, Germany) used in this work have a glass transition temperature (T_g_) of 142 °C with a thickness of 1.5 mm. The COC structures were fabricated by hot embossing in a hydraulic press (25T Hydraulic Lamination Hot Press, MTI Corporation, Richmond, CA, USA), as represented in Figure 1A. A stack composed of an aluminium plate, a thin layer of PDMS to obtain a clear backside in the COC, an aluminium frame containing the COC substrate and the epoxy mould with the structures facing down, and an aluminium block (Figure 1B) was placed between the press plates. The aluminum frame allows for the processing of up to six COC substrates. The embossing process occurs for 10 min, at 160 °C and 2.3 MPa. After 10 min of embossing, the press temperature was turned off and the stack was maintained between the plates for another 15 min. After this time, the pressure was released and the embossed COC substrate was removed, completing the de-embossing step.

The height of the embossed COC structures was measured using a profilometer (AlphaStep 200 Profilometer, KLA Corporation, Milpitas, CA, USA).

#### 2.1.6. Sealing and Connections

The inlets and outlets in the COC substrates were drilled before sealing using a MiniTech milling machine (Minitech Machinery Corp, Norcross, GA, USA).

The embossed COC substrates were sealed against a 1.5 mm thick COC plate by thermal bonding. First, the COC substrates were cleaned with IPA and dried; next, the substrates were assembled between two PDMS slabs and inserted in the hydraulic press for 30 min at 130 °C and 2.3 MPa.

The connections with external fluidic sources were performed with PEEK commercial connectors (N-333 NanoPort, microfluidic ChipShop GmbH, Jena, Germany) fixed to the substrate with a 2-part hybrid adhesive (LOCTITE^®^ HY4090, Henkel AG & Co. KGaA, Düsseldorf, Germany). The connectors are clamped to the structure for at least 12 h to ensure that there are no leaks between the ports and the structures.

### 2.2. Methods of Experimental Assays of COC Structures

#### 2.2.1. Hydrophilization Surface Treatments

All substrates were embossed before the treatments. In the first treatment, the samples were placed in the UVO cleaner (28–32 mW/cm² at 253.7 nm) for 25 min. In a second treatment, the samples were placed in an ultrasonic bath (Kerry Pulsatron MKC22, Guyson, Skipton, North Yorkshire, UK) for 2 min with acetone. In the third treatment, the samples were placed in the oxygen plasma cleaner for 4 min at high intensity (30 W). The fourth treatment consisted of placing the samples in the ultrasonic bath for 2 min with acetone, followed by oxygen plasma for 4 min at high intensity. In the fifth treatment, 2 mL of a mixture of 35% cyclohexane and 65% acetone (*v*/*v*) was poured onto the sample and kept there for 90 s.

#### 2.2.2. Hydrophilization Surface Treatments—Contact Angle Measurements

A water droplet of known volume (20 µL) was placed on the surface of the sample and photographed perpendicular to the surface with a digital camera (Samsung WB100, Samsung Group, Suwon, Republic of Korea). A stand was used to capture the image, where the samples were at a height of 3 cm, and the digital camera was placed 12 cm away from the sample. The images obtained were analysed using the imaging software ImageJ 1.47 (NIH, Bethesda, MD, USA) with the Drop Shape Analysis plugin [29,30].

#### 2.2.3. Burst Pressure Tests in Microfluidic Structures

Burst pressure tests were performed with compressed air both on COC and PDMS devices. The microfluidic structures used had only the inlet open. In the pressurization tests using compressed air in the COC substrates, the inlets had PEEK connectors with tubing connected to a pressure regulator (IR2010-F02, SMC Corporation of America, Noblesville, IN, USA). In PDMS structures, the PEEK connector was fixed with PDMS, while in the COC, it was fixed with Loctite HY4090 adhesive. With the compressed air on, an increasing pressure between 0.1 MPa and 0.4 MPa was applied until the rupture of the microfluidic structure was observed.

#### 2.2.4. Temperature Tolerance Tests

The temperature tolerance tests were performed in COC and PDMS microfluidic structures filled with aqueous solutions with food colouring to observe the formation of bubbles. The aqueous solutions were degassed in an ultrasonic bath for 15 min prior to insertion in the microfluidic structure. This experiment consisted of heating the microfluidic structure on a hot plate at 95 °C while flowing the liquid with a syringe pump (NE-1002X, New Era Pump Systems, Inc., Farmingdale, NY, USA) at 1 µL/min. A control experiment was also performed at room temperature (~25 °C).

#### 2.2.5. Molecular Diffusion Measurements

Molecular diffusion into the walls of the microfluidic structures (COC and PDMS) was studied using a rhodamine B solution (Rhodamine B base, dye content 97%, Sigma-Aldrich, St. Louis, MO, USA) (1 mg/mL) and fluorescence images were acquired with an inverted fluorescence microscope (CKX41 Inverted Microscope, Olympus Corporation, Shinjuku, Tokyo, Japan) coupled to a charge-coupled device (CCD) colour camera (XC30, Olympus Corporation, Shinjuku, Tokyo, Japan). The microscope was also equipped with a filter cube with a band-pass excitation of 480–550 nm and a long-pass emission of 590 nm (Excitation filter BP480-550C, Olympus Corporation, Shinjuku, Tokyo, Japan).

In these experiments, the channel was filled with the fluorophore solution using a syringe pump to completely fill the channel. Images were acquired every 2 min for 1 h with an exposure time of 50 ms. The fluorescence intensity was quantified using the ImageJ software.

#### 2.2.6. Proof-of-Concept Assay: DNA Capture on Streptavidin Beads and cDNA Hybridization

The streptavidin agarose beads used in these assays were purchased from EMD Millipore Corp USA, Merck kGaA (Darmstadt, Germany). The DNA sequences were purchased from Integrated DNA Technologies (Leuven, Belgium) and Stabvida Genomics Lab (Caparica, Portugal) and stored in 100 μM aliquots at −20 °C and are presented in Table 2.

Before the assay, the streptavidin beads were inserted into the microchannel by injecting a solution of beads diluted in ethanol 20% at 5 µL/min, followed by a wash with PBS 1X at 20 µL/min.

The proof-of-concept assay was performed by flowing a solution of 100 nM of primer, 250 nM of padlock, and 250 nM of Atto-430LS labelled complementary target ssDNA at 0.5 µL/min for 40 min through the microchannel that had previously been loaded with the functionalized beads. This was followed by a wash to remove any unbound molecules with PBS 1X at 5 µL/min. To evaluate the specificity of the assay, a negative control was performed using a labelled non-complementary ssDNA strand, as well as a control with no biotinylated primer bound to the beads.

End-point fluorescence measurements were taken using a fluorescence microscope (Leica DMLM, Leica Microsystems, Wetzlar, Germany) coupled to a CCD colour camera (Leica DFC300 FX, Leica Microsystems, Wetzlar, Germany) and a 100 W mercury short-arc lamp and an I3 blue excitation filter cube with a band-pass excitation of 450–490 nm and a long-pass emission of 510 nm. The fluorescence intensity was quantified using the ImageJ software.

## 3. Results and Discussion

### 3.1. COC Structures Fabrication

#### 3.1.1. Embossing and De-embossing

During the development of the COC embossing process, the stack composition, the embossing temperature, and the embossing time were optimized. When COC substrates are heated above the glass transition temperature (T_g_) they soften [11] and take on the features of the objects with which they are in contact. For this reason, the COC substrates were embossed using the stack represented in Figure 1A. The bottom part of the stack has an aluminium plate covered with a thin film of PDMS to allow the transfer of PDMS features while having a clear backside. The aluminium frame, containing the negative PDMS mould and the COC substrate, is placed on top of the PDMS-coated aluminium plate. On the top of the stack, an aluminium block is placed to allow a uniform pressure distribution along the stack. Since PDMS is an elastomer, the application of pressure in the embossing process led to some deformation of the COC substrates creating round edges and also deformations in the embossed features, with height loss relative to the original mould. These challenges led us to explore rigid substrates, such as epoxy, which allows the complete replication of the desired features. During the embossing, due to the expansion of COC when heated and pressed [23], the frame was fixed with Kapton tape to prevent the movement of the stack.

**Figure 2:**
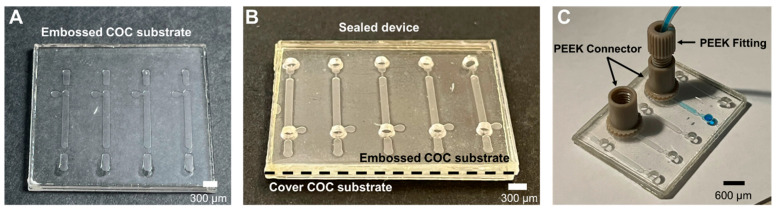
Microfluidic structures processed in COC photographed after each step of the process. (**A**) Shaping the microchannels through embossing (160 °C, 10 min, 2.3 MPa); (**B**) sealing using thermal bonding (130 °C, 30 min, 2.3 MPa); and (**C**) connecting the microfluidic device to the outside world using PEEK connectors glued to the device.

After the stack composition was optimized, it was necessary to adjust the embossing conditions. The first embossing temperature tried was 170 °C because it is more than 20 °C above the T_g_ of the COC used (T_g_ = 142 °C) [31,32]. The de-embossing time was fixed first at 30 min because after this time the press temperature is around 110 °C, which is below the T_g_ of the COC, thus allowing the substrates to solidify. The value for the applied pressure was fixed at 2.3 MPa. At the start of the optimization, the COC substrates were pressed for 1 h at 170 °C, but the resulting structures were deformed and included trapped air bubbles, due to the high temperature applied. This observation led to the testing of lower embossing temperatures, varying between 140 °C and 170 °C at intervals of 10 °C, while keeping the embossing pressure constant at 2.3 MPa. The embossing time was tested between 10 and 30 min, at intervals of 10 min, while using the above-mentioned range of temperatures. At the end of the embossing tests, the best results, defined by the channels that upheld the designed dimensions, were obtained using an embossing time of 10 min and an embossing temperature of 160 °C. In summary, the final optimized embossing process consists of applying a pressure of 2.3 MPa for 10 min at 160 °C, while the de-embossing time is kept at 15 min. Using this aluminum frame, it was possible to process up to six COC substrates at the same time. If there is a change in the frame, it could be possible to process a large number of devices simultaneously. Figure 2A represents an embossed COC substrate with microfluidic channels, applying the process described earlier.

The height of the embossed features was measured with a profilometer. After measuring the COC substrates embossed under the optimal conditions, several other embossing times (5, 20, and 30 min) were also tested to see if improvements could be observed in the feature transfer quality. No significant difference in height was observed when the embossing times were changed from 5 to 30 min. When changing to higher embossing temperatures both the COC substrate and the epoxy mould show deformations in the features (Table 3). Substrates embossed at lower temperatures start having a significant loss of height (Table S1). Using the embossing process at 160 °C, the substrates are replicated with ~98% of the height of the original structure in the mould (Table S2). The smaller features replicated using this method had a height of 10 µm and width of 10 µm. The surface roughness of the microchannel was also measured in the profilometer, obtaining an average of 1.12 ± 0.72 µm.

#### 3.1.2. Sealing

The method chosen to seal the embossed channel was thermal bonding, using another COC substrate as cover. In thermal bonding, the substrates need to be in contact with each other under certain conditions (pressure, temperature, and time) to allow the diffusion of the polymer molecules between the two substrates, resulting in a strong bond [33]. First, temperatures above T_g_ (150 °C and 160 °C) were tested while the value of applied pressure was fixed to 2.3 MPa, but these resulted in weak bonds when pressed for short times (10 min or less), and resulted in collapsed microchannels if more time was used. This observation led us to lower the embossing temperatures, instead of using temperatures above T_g_. In the literature, several methods that use temperatures below the T_g_ are reported but all of those examples require some surface treatment before the sealing, such as UVO [34], solvents [35], or plasma treatments [36]. These treatments are administered to the surface of the thermoplastic and result in a decreased T_g_ only at the surface, allowing a low-temperature thermal bonding without deformation of the structures. Before resorting to the use of these treatments, we tried to reduce the temperature to 140 °C while maintaining the 10 min of bonding time. At 140 °C the structures collapsed, so temperatures between 110 °C to 135 °C were tested. Temperatures below 130 °C resulted in a non-uniform and weak bond, with very different results obtained. At 135 °C the features collapsed. At 130 °C a uniform bond was obtained, but the bond strength had to be evaluated. To evaluate the bond strength, the bonding time was tested from 10 to 30 min, while fixing the temperature at 130 °C and the pressure at 2.3 MPa, and a simple delamination test was performed with the help of tweezers [37]. The structures sealed for 10 min had a weak bond and it was easy to peel the substrates, while the substrates bonded for 30 min offered much more resistance to separation (Table 4). Using 130 °C and 2.3 MPa for 30 min resulted in a strong bond capable of withstanding high pressures. An example of a sealed device can be observed in Figure 2B.

Another approach tried was the use of solvent bonding. In this case, instead of another COC substrate, a COC film with a thickness of 135 µm and T_g_ = 78 °C was used. The method developed by Keller et al. [38], in which they use a mixture of 35% cyclohexane with 65% acetone (*v*/*v*) to seal the channels, was adapted. In this method, the membrane was exposed to the solvent mixture, cleaned with acetone, and followed by the application of uniform pressure. This method can seal embossed COC structures without deforming them, but the exposure to the solvent mixture was not uniform, resulting in white stains appearing in the membrane and impairing the device transparency. The white stains were associated with a higher exposure to the solvent solution and consequently a more dissolved membrane. For this reason, and because the T_g_ of the membrane is low, the thermal bonding method was selected.

#### 3.1.3. Connections

The COC substrates are rigid, meaning that just inserting a metallic adapter in the inlets as is done with the elastomeric PDMS will result in leaks around the metallic adapter. For rigid substrates, one of the available options is to glue the fluidic connections to ensure that they are tight. One of the main goals of this work is to develop COC-based chips that can withstand high pressures for future chromatography applications and resist high temperatures for DNA denaturation experiments, which is why the nanoports were chosen. The PEEK connectors are used in many chromatography apparatuses and can be used at temperatures up to 250 °C.

The nanoports used have a footprint of 8.4 mm and an internal diameter of 1.6 mm. In the developed device, the holes had a nominal diameter of 1.5 mm, but these types of connections can have smaller diameters. The Loctite HY4090 sealant adhesive is suitable for use in plastics and is transparent and colourless, being ideal for this type of application. The nanoports are fixed by placing the adhesive around the edge of the adaptor and inserting them around the hole. After about 30 min, the nanoports are firmly in place, but to ensure better adhesion to the substrate, it is recommended to wait at least 12 h before use. Using glue to obtain tight fluidic connections may result in the clogging of the inlets with glue, and the user must be aware of this and ensure that the glue does not enter the device channels. A complete COC device with nanoports is shown in Figure 2C.

### 3.2. Hydrophilization Surface Treatments

After the fabrication process of the COC microfluidic structure was completed, several surface treatments were tested on the substrates and membranes to control their hydrophilicity. Hydrophilic behaviour would enable the devices to be used in, for example, capillary applications without the need to use external sources for pumping, and to decrease protein adsorption in biosensing applications. For the surface to be considered hydrophilic, it needs to have a contact angle lower than 90° [39], but it is advisable that it be smaller than 60° for better performance [40]. The surface will then create a concave interface allowing fluid movement inside the channel [40].

**Figure 3:**
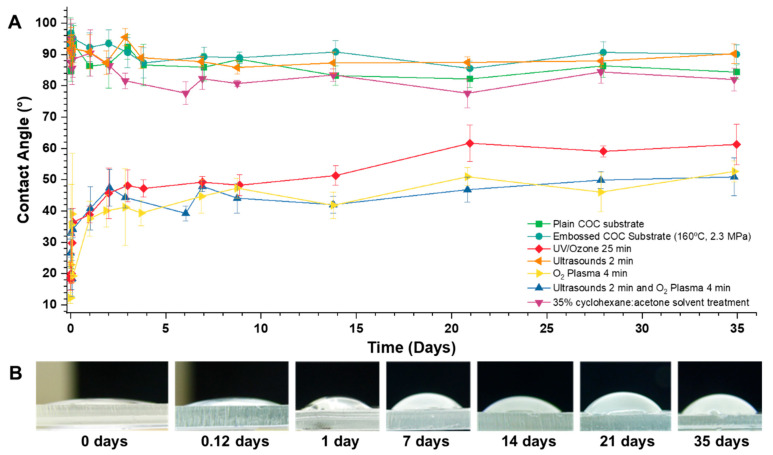
(**A**) Graphic representation of contact angles with water measured on COC substrates for untreated COC (green squares), for untreated embossed COC (turquoise circles), and after treatment with UVO (red diamond), ultrasonic bath (orange left triangle), oxygen plasma (yellow right triangle), ultrasonic bath with oxygen plasma (blue up triangle), and treatment with 35% cyclohexane with 65% acetone (*v*/*v*) mixture (purple down triangle). Each point was measured in triplicate, and error bars correspond to ± standard deviation (SD). (**B**) Close view of oxygen plasma effect on the contact angle with water of the COC surface over 35 days.

The treatments tested to render the COC surface hydrophilic consisted of: UVO cleaning, ultrasonic bath, oxygen plasma, ultrasonic bath followed by oxygen plasma, and a mixture of 35% cyclohexane with 65% acetone (*v*/*v*). The effect of the treatments in the surface hydrophilization was monitored for 35 days by measuring the contact angles using a sessile drop technique. As control samples, the contact angles of an untreated substrate before and after embossing were measured, indicating that the contact angle of a regular COC substrate is ~90° ± 3. To establish a baseline, first contact angles were measured on COC substrates that were previously embossed. The results obtained for the contact angle of water from the different treatments made on the COC substrate surfaces can be seen in Figure 3A. When the surface was exposed for 25 min to UVO, a decrease from 95° to 19° in the contact angle was observed shortly after exposure. The same behaviour occurred as a result of the treatment with oxygen plasma and the treatment with an ultrasonic bath followed by oxygen plasma. These three treatments showed a low contact angle after exposure (~12–18°) which increased with time and started to stabilize after seven days. This increase in contact angle continued during the 35 days of monitoring, with the final, stabilized contact angles being 61.27° ± 4 for UVO treatment, 52.65° ± 3 for oxygen plasma treatment, and 50.91° ± 3 for the treatment with an ultrasonic bath followed by oxygen plasma. The increasing contact angles for the oxygen plasma treatment can be visualized in the photographs shown in Figure 3B. The surface exposed only to the ultrasonic bath showed no signs of hydrophilicity, maintaining a contact angle similar to the control sample. The treatment in which the COC surface was exposed to the cyclohexane:acetone mixture also showed no hydrophilic behaviour, with a contact angle of water identical to the control samples.

### 3.3. Burst Pressure Tests in Microfluidic Structures

**Figure 4:**
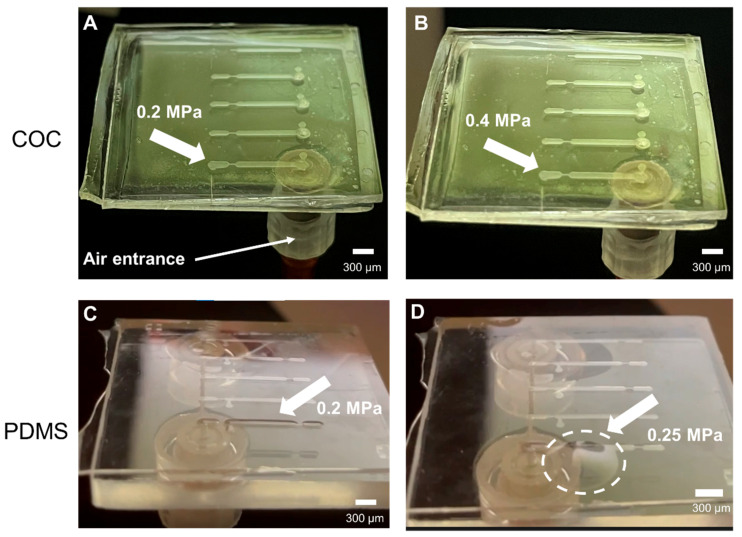
Photographs obtained during burst pressure tests in COC and PDMS microfluidic devices. The pressurized channels are indicated by the white arrows. (**A**) COC device with 0.2 MPa and no deformation observed in the channel; (**B**) COC device with 0.4 MPa and still no deformation observed in the channel; (**C**) PDMS device with 0.2 MPa and swollen channel; and (**D**) PDMS device with 0.25 MPa with an unsealed channel due to the high applied pressure.

Burst pressure tests were used to evaluate how much pressure the fabricated COC microfluidic devices can withstand without rupture. The experiment started by applying a pressure of 0.1 MPa for 5 min, and if the device could withstand the applied pressure, the pressure was raised in 0.1 MPa steps until the pressure reached 0.4 MPa. The COC device was able to withstand 0.4 MPa with no leakage or deformation detected, as can be seen in both Figure 4A, which shows the device at 0.2 MPa, and Figure 4B, which shows the device at 0.4 MPa. The same procedure was applied in PDMS devices. Up to 0.2 MPa, the PDMS channel swells but stays sealed, as observed in Figure 4C. Even though the PDMS device is still sealed, the swelling of the channel will affect the internal volume of the channel, and in the case of microbeads being used, these would be rearranged, or even escape the height trap, affecting the whole assay. Since it is reported in the literature that PDMS devices bonded with oxygen plasma might burst if a higher pressure is applied [37], the burst pressure was increased. At 0.25 MPa, the device could not withstand the pressure, unsealing the device, sometimes tearing the PDMS membrane, as observed in Figure 4D.

Although the maximum pressure tested was 0.4 MPa, COC devices are expected to withstand even higher pressures. In several articles it is mentioned that the COC devices can withstand pressures such as 15.6 MPa [41] and 34.6 MPa [42], depending on the method and parameters used for bonding. The high pressures are mainly needed for chromatographic applications, while the 0.4 MPa is sufficient for general applications where the flows required are within a few microliters per minute (0.1–40 µL/min).

### 3.4. Temperature Tolerance Tests

The temperature tests were used to assess the material permeability to gases and the resistance of the devices to high temperatures. These tests were performed at 95 °C, which is a temperature used for PCR applications, with PDMS being used for comparison. A control experiment was performed at room temperature.

**Figure 5:**
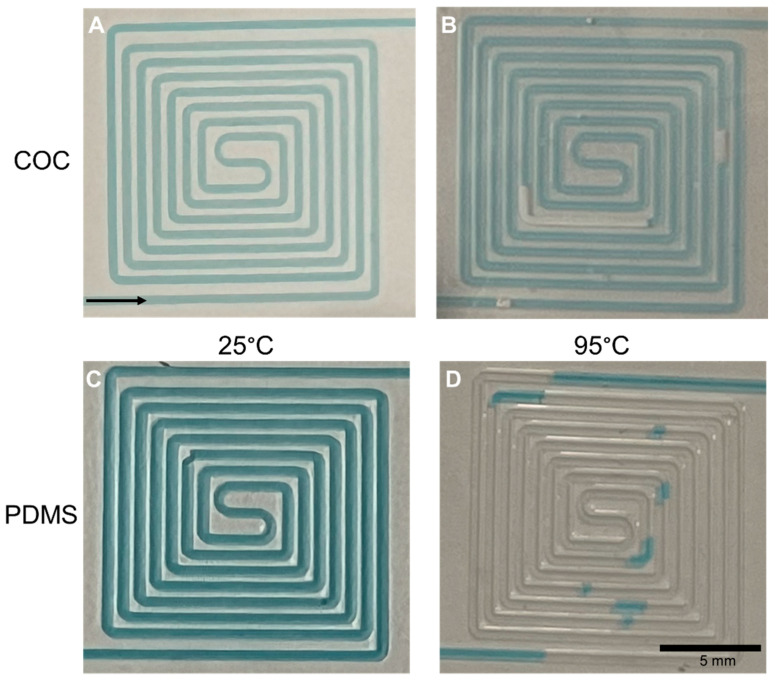
Photographs of the aqueous solution flowing through the COC and PDMS devices at different temperatures. (**A**) COC device at 25 °C with no bubbles formed; (**B**) COC device at 95 °C with some bubbles observed inside the channel; (**C**) PDMS device at 25 °C with no bubbles formed; and (**D**) PDMS device at 95 °C, with a large formation of bubbles observed along the channel. The black arrow indicates the direction of the flow.

The hot plate was set to the desired temperature, and the aqueous solution flowed through the channel at 1 µL/min. At 25 °C, both the COC and PDMS channels showed no bubble formation, as seen in Figure 5A,C respectively. At 95 °C, the COC has a constant flow through the channel, with low formation of bubbles (Figure 5B). The small formation of bubbles seen in COC happens due to insufficient degassing of the liquid, and possibly small deformations inside the microfluidic channel. Even though these bubbles are formed, they still go through the channel along with the liquid. This happens due to the very low oxygen permeability in COC which prevents the evaporated liquid from leaving the channel. It was also observed that these bubbles were always formed in the same zones, which means that the formation of bubbles might happen due to small deformations or defects inside the microfluidic structure.

In the PDMS device heated to 95 °C, it is possible to observe a large formation of bubbles along the full length of the channel (Figure 5D). These bubbles are constantly formed due to the high oxygen permeability of PDMS, allowing the appearance of empty spots through the channel, which pulls the liquid faster in some zones. Even though the liquid flow was constant in both materials, the solution reached the end of the PDMS structure first due to the presence of the air gaps.

The results observed are in accordance with the literature that previously reported the formation of air bubbles in heated PDMS [43,44] and the evaporation of fluids at high temperatures [45]. The gas permeability in COC, at 35 °C, is reported to be 2.55 barrer [46]. With this low gas permeability, the gas bubbles present do not pass through the COC material and will eventually leave the channel carried by the flowing fluid. In PDMS, the gas permeability is reported to be between 523 barrer [47] and 733 barrer [48] at 35–37 °C. In PDMS, it was observed that bubbles formed continuously through the entire length of the channel. Due to the high gas permeability of PDMS, the air trapped inside the channel can pass through the material, but it can also be renewed due to the device being in contact with air, allowing a continuous formation of bubbles.

### 3.5. Molecular Diffusion Measurements

**Figure 6:**
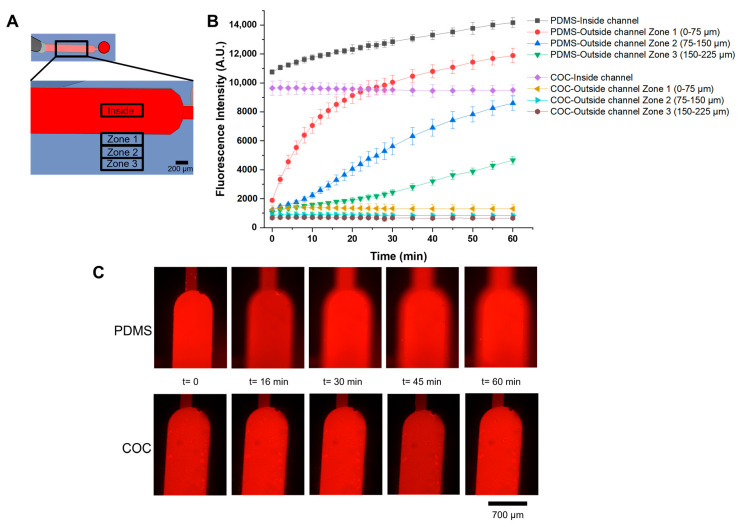
(**A**) Zones for fluorescence measurements in the molecular diffusion experiment. The defined zones were inside the channel, outside the channel walls in zone 1, between 0 and 75 µm (with the zero being in the margin of the channel), zone 2, between 75 and 150 µm, and in zone 3, between 150 and 225 µm from the channel wall. (**B**) Representation of fluorescence intensity over time of rhodamine B in PDMS and COC. Each point was measured in triplicate and error bars correspond to ± SD. (**C**) Increase in fluorescence intensity over time in PDMS (**top**) and in COC (**bottom**). The images were acquired with an exposure time of 50 ms and 0 dB gain.

Molecular diffusion in PDMS devices is considered a problem [14,16]. The molecules—in particular, molecules with hydrophobic characteristics, which is the case of several biomolecules such as proteins and hormones–present in the solution flowing in the PDMS channels can penetrate into the PDMS, diffusing to some extent. The diffusion of molecules will change their concentration in the microchannels and chambers of the microfluidic devices, possibly compromising the assays. It is thus important to verify the solid phase diffusion behaviour of molecules in COC microchannels. A diffusion experiment was designed to assess the diffusion of specific molecules to the bulk for the different microfluidic device materials. A rhodamine B solution was pumped into the channel, and the fluorescence was monitored continuously for 1 h. During the fluorescence measurement the flow was stopped, so that the volume and initial concentration of rhodamine B inside the channel were kept constant. The fluorescence analysis was performed in four different zones: inside the channel, and outside the channel walls in three defined areas successively further away from the channel: zone 1, between 0 and 75 µm (with the zero being in the margin of the channel), zone 2, between 75 and 150 µm, and in zone 3, between 150 and 225 µm from the channel wall (Figure 6A). The results obtained after analysing the fluorescence images are represented in Figure 6B.

The diffusion of rhodamine B has a considerable range inside the PDMS microchannel walls. In Figure 6A, it is possible to observe the increase in fluorescence over time because rhodamine B diffusion in the PDMS occurs isotropically, also entering the upper part of the PDMS. The farther the zone is from the channel, the smaller the increase in fluorescence intensity. In zone 1, the fluorescence intensity is very close to that of the zone inside the channel, and zone 3 marks the end of the diffusion area for the time and concentrations used in this test, so it has a relatively low fluorescence signal. The respective images of the diffusion over time in the PDMS channel and the COC channel can be seen in Figure 6C. In COC, the signal inside the channel and in zones 1, 2, and 3 did not change throughout the experiment, and no diffusion was observed, indicating that COC was not permeable to rhodamine B, and suggesting that it should be a more suitable material for biological experiments such as biosensing and organ-on-a-chip devices.

### 3.6. Proof-of-Concept Assay: DNA Capture on Streptavidin Beads and cDNA Hybridization

To validate the COC microfluidic device, a proof-of-concept assay was performed to capture a labelled target ssDNA to demonstrate its functionality to perform miniaturized assays. To trap the beads inside the microfluidic channel, a two-height structure was fabricated consisting of a 100 µm height channel, followed by a smaller 20 µm height channel that will trap the beads where the assay takes place [49].

**Figure 7:**
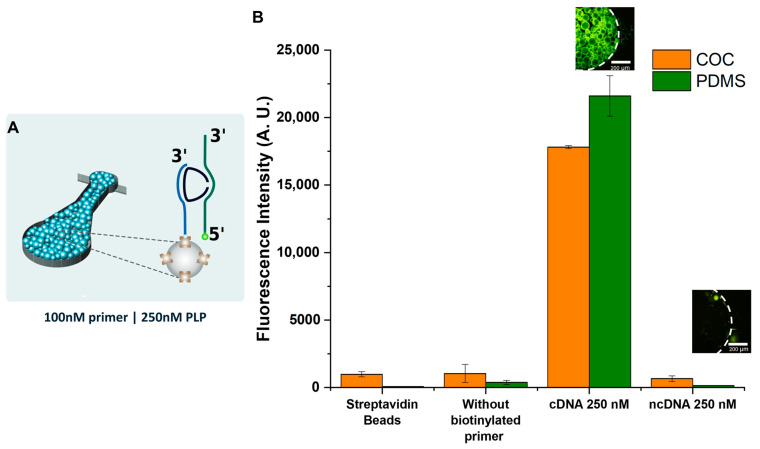
(**A**) Schematic representation of the proof-of-concept assay (the biotinylated primer is represented in light blue, the padlock is represented in dark blue, and the single-stranded target DNA labelled with Atto430-LS is represented in green). (**B**) Representation of fluorescence measurements obtained for COC (orange) and PDMS (olive) devices (each point was measured in duplicate, and error bars correspond to ± SD), and microscope images obtained for COC devices (the images were acquired with an exposure time of 1 s and 1× gain). The images were contrast-enhanced for better visualization.

In this assay, shown in Figure 7A, the channel was packed with streptavidin microbeads (~90 µm diameter), and then a solution containing the biotinylated primer, the padlock and the labelled complementary target ssDNA flowed through the microchannel containing the beads. In the negative control, instead of the complementary target ssDNA, a labelled non-complementary target ssDNA was used. To determine if any of the labelled ssDNA was non-specifically binding to the beads, a control with no biotinylated primer bound to the beads was performed. The assays were also performed on PDMS microchannels for comparison.

The results obtained are presented in Figure 7B. To obtain a baseline, the fluorescence signal of a channel packed with streptavidin beads was measured, confirming that the beads do not fluoresce and that the baseline fluorescence is low. This assay is based on the biotin–streptavidin bond of the beads and the primer. The biotinylated primer binds to the streptavidin beads, followed by the hybridization to the back of the padlock. The labelled target ssDNA is complementary to the ends of the padlock, allowing its hybridization. The biotin–streptavidin interaction in the assay was tested by removing the biotinylated primer to determine if the labelled DNA would non-specifically bind to the streptavidin beads or the channel walls, which did not happen. Lastly, the positive assay, using the labelled target ssDNA strand, and the negative assay, using a labelled non-complementary ssDNA strand, were performed, detecting the complementary ssDNA, but having a very low fluorescent signal for the non-complementary ssDNA, proving the specificity of the assay. The results obtained for the COC microfluidic device were similar to those obtained for an equivalent PDMS device.

## 4. Conclusions

In this work, a rapid and scalable method for the fabrication of microfluidic biochips using COC was developed. This method uses hot embossing to imprint features in the COC substrate, thermal bonding to seal the substrates, and commercial PEEK connectors to interact with external fluidic sources. Complete microfluidic devices with several embossed features with ~98% of the height of the original structure in the mould were achieved. The COC microfluidic devices showed higher pressure resistance, less bubble formation at high temperatures, and less molecular diffusion into the bulk of the material than PDMS microfluidic devices. In a proof-of-concept biosensing assay, the COC microfluidic devices were shown to produce similar results to the ones obtained with the standard PDMS device.

## Figures and Tables

**Table 1:** Comparison of several hot embossing methods described in the literature.

COC Type	T_embossing_	P_embossing_	t_embossing_ (min)	Bonding Lid	Bonding Method	Reference
Substrate—Topas, 1.5 mm thick, T_g_ = 142 °C	160 °C	2.3 Mpa	10	Substrate—Topas, 1.5 mm thick, T_g_ = 142 °C	Thermal bonding: 130 °C; 30 min; 2.3 Mpa	This paper
Substrate—Zeonor, 2 mm thick, T_g_ = 105 °C	130 °C	250 psi	7	Substrate—Zeonor, 2 mm thick, T_g_ = 105 °C	Thermal bonding: 85 °C; 10–15 min; 200 psi	[26]
Substrate—Zeonor, T_g_ = 100 °C and T_g_ = 102 °C	125 °C	3400 kg load	10	Substrate—Zeonor, T_g_ = 100 °C and T_g_ = 102 °C	Thermal bonding: 96 °C; 20 min; 2300 kg load	[27]
Substrate—Topas, T_g_ = 130 °C	Heated under vacuum up to 175 °C and embossed at 175 °C.	3 kN	5	Composite foil made of Topas 8007 (T_g_ = 75 °C) and Topas 6013 (T_g_ = 130 °C) (annealed at 75 °C for 1 h)	Thermal bonding in a lamination machine: two cylinders heated up to 120 °C, with a pressure of 5 bar, and a feed rate of 30 cm/min.	[25]
Substrate—Topas, 1 mm thick, T_g_ = 160 °C	170 °C	2.94 kN	3	Substrate—Topas, 1 mm thick, T_g_ = 130 °C	Thermal bonding: 125 °C; 6 min; 0.5 kN	[28]
Substrate—Topas, 1 mm thick, T_g_ = 160 °C	170 °C	2.94 kPa	1 h for annealing and 4 min for embossing.	Substrate—Topas, 1 mm thick, T_g_ = 160 °C	Thermal bonding: 150 °C, 160 °C, and 170 °C; 6 min; 2 Mpa.	[24]

**Table 2:** Summarized list of the oligonucleotide sequences used in this work, as well as their modifications (mod).

Oligonucleotides	Sequence (5′-3′)	5′mod	3′mod
Primer	TTTTTTTTTTGTAAGACAC TATTACTGAGGA	Biotin	None
Padlock	TGCTTTGTTTCAGGTGTAG TGTATGCAGCTCCTCAGT AATAGTGTCTTACGGCAT CACTGGTTACGTCTGTCT CTACACCTTTTTTAGGA	PO4-phosphorilation	None
Complementary target ssDNA	TTAAATTAATGTACAAAGG TCAACCAATGACATTCAGA CTATTATTGGTTGATACAC CTGAAACAAAGCATCCTA AAAAAGGTGTAGAGA	Atto-430LS	None
Non-complementary target ssDNA	CGTGTCGTTCACATCTGT CCGT	Atto-430LS	None

The section highlighted in blue in the primer and padlock sequence, as well as the sections highlighted in red in the padlock and complementary target ssDNA represent a complementary hybridization area.

**Table 3:** Summary of embossing parameters and results obtained in COC.

T_embossing_ (°C)	P_embossing_ (MPa)	t_embossing_ (min)	T_de-embossing_ (min)	Observations
170	2.3	60	30	Deformed COC structures and deformed epoxy mould
160	2.3	5/10/20/30	15	Successfully embossed COC structures at all times (98% height replication at 10 min)
150	2.3	10/20/30	15	Embossed COC structures with 60% height replication
150	2.3	5	15	Non-uniform embossing
140	2.3	5/10/20/30	15	No embossing observed

**Table 4:** Summary of sealing conditions tested in embossed COC structures.

T_sealing_ (°C)	P_sealing_ (MPa)	t_sealing_ (min)	Observations
150/160	2.3	<10	Unsealed structures with non-uniform strong bonds
150/160	2.3	>10	Collapsed microchannels
140	2.3	10	Collapsed microchannels
110/120	2.3	10	Unsealed structures with non-uniform weak bonds, easy to delaminate
135	2.3	10	Collapsed microchannels
130	2.3	10/20	Sealed structure with uniform bond
130	2.3	30	Sealed structure with uniform bond and strong resistance to delamination

## Data Availability

Not applicable.

## Supplementary Materials

The following supporting information can be downloaded at: https://www.mdpi.com/article/10.3390/mi14101837/s1, Table S1: Height of COC microchannels embossed at 150 °C for 30 minutes. Each channel was measured in the epoxy master mould and in two embossed COC substrates. Table S2: Height of COC microchannels embossed at 160 °C for 10 minutes. Each channel was measured in the epoxy master mould and in two embossed COC substrates.
