# Supplementary material for: Scalable Processing of Cyclic Olefin Copolymer (COC) Microfluidic Biochips
Source: Micromachines (Basel). 2023 Sep 27;14(10):1837. doi: 10.3390/mi14101837 (PMC10609239; doi:10.3390/mi14101837)
Supplement: Supplementary file 1 [file micromachines-14-01837-s001.zip › micromachines-2624407-supplementary.pdf]

Supplementary Information

Table S1. Height of COC microchannels embossed at 150 °C for 30 minutes. Each channel was measured in the epoxy master mould and in two embossed COC substrates.

Channel	1	2	3	4	5
			Height (μm)		
**Epoxy**	91.14	86.28	85.66	100.9	117.4
**COC 1**	51.8	55.09	51.74	59.64	73.63
**COC 2**	52.84	47.85	50.09	55.72	69.32

Table S2. Height of COC microchannels embossed at 160 °C for 10 minutes. Each channel was measured in the epoxy master mould and in two embossed COC substrates.

Channel	1	2	3	4	5
			Height (μm)		
**Epoxy**	121.3	114.9	127.7	119.4	115.4
**COC 1**	120.1	115.1	127.2	118.6	110.9
**COC 2**	116.4	114.3	123.2	118.2	106.6
